# Tissue-derived mesenchymal stromal cells used as vehicles for anti-tumor therapy exert different *in vivo* effects on migration capacity and tumor growth

**DOI:** 10.1186/1741-7015-11-139

**Published:** 2013-05-28

**Authors:** Carolina Belmar-Lopez, Gracia Mendoza, Daniel Oberg, Jerome Burnet, Carlos Simon, Irene Cervello, Maite Iglesias, Juan Carlos Ramirez, Pilar Lopez-Larrubia, Miguel Quintanilla, Pilar Martin-Duque

**Affiliations:** 1 Instituto Aragones de Ciencias de la Salud-IIS Aragon, Zaragoza, Spain; 2 Department of Medical Biochemistry and Microbiology, The Biomedical Centre, Uppsala University, Uppsala, Sweden; 3 Queen Mary, University of London/Cancer Research UK, London, UK; 4 Fundacion IVI, Instituto Universitario IVI, University of Valencia, Institute of Health Research INCLIVA, Valencia, Spain; 5 Facultad de Ciencias Biosanitarias, Universidad Francisco de Vitoria, Madrid, Spain; 6 Centro Nacional de Investigaciones Cardiovasculares, Madrid, Spain; 7 Instituto de Investigaciones Biomedicas Alberto Sols (CSIC-UAM), Madrid, Spain; 8 Fundacion Araid, Zaragoza, Spain

**Keywords:** Mesenchymal stromal cells, Migration, In vivo imaging, Tumor growth, Pluripotency

## Abstract

**Background:**

Mesenchymal stem cells (MSCs) have been promoted as an attractive option to use as cellular delivery vehicles to carry anti-tumor agents, owing to their ability to home into tumor sites and secrete cytokines. Multiple isolated populations have been described as MSCs, but despite extensive *in vitro* characterization, little is known about their *in vivo* behavior.

The aim of this study was to investigate the efficacy and efficiency of different MSC lineages derived from five different sources (bone marrow, adipose tissue, epithelial endometrium, stroma endometrium, and amniotic membrane), in order to assess their adequacy for cell-based anti-tumor therapies. Our study shows the crucial importance of understanding the interaction between MSCs and tumor cells, and provides both information and a methodological approach, which could be used to develop safer and more accurate targeted therapeutic applications.

**Methods:**

We first measured the *in vivo* migration capacity and effect on tumor growth of the different MSCs using two imaging techniques: (*i*) single-photon emission computed tomography combined with computed tomography (SPECT-CT), using the human sodium iodine symporter gene (hNIS) and (*ii*) magnetic resonance imaging using superparamagnetic iron oxide. We then sought correlations between these parameters and expression of pluripotency-related or migration-related genes.

**Results:**

Our results show that migration of human bone marrow-derived MSCs was significantly reduced and slower than that obtained with the other MSCs assayed and also with human induced pluripotent stem cells (hiPSCs). The qPCR data clearly show that MSCs and hiPSCs exert a very different pluripotency pattern, which correlates with the differences observed in their engraftment capacity and with their effects on tumor growth.

**Conclusion:**

This study reveals differences in MSC recruitment/migration toward the tumor site and the corresponding effects on tumor growth. Three observations stand out: 1) tracking of the stem cell is essential to check the safety and efficacy of cell therapies; 2) the MSC lineage to be used in the cell therapy needs to be carefully chosen to balance efficacy and safety for a particular tumor type; and 3) different pluripotency and mobility patterns can be linked to the engraftment capacity of the MSCs, and should be checked as part of the clinical characterization of the lineage.

## Background

Human mesenchymal stem cells or mesenchymal stromal cells (MSCs) are multipotent progenitor cells or adult stem cells that exhibit the ability to migrate and engraft into tumor sites when delivered systemically [1]. However, determining the most appropriate clinical application of MSCs is hampered by the current lack of knowledge about how these cells behave *in vivo.* The precise mechanisms behind the recruitment of MSCs to tumor sites and their migration across the endothelium are not yet fully understood. It is probable that damaged tissue expresses specific receptors or ligands to make possible trafficking, adhesion, and extravasation of MSCs to the site of damage and recruitment to inflammation sites, using a mechanism similar to leukocyte migration [2-4].

The most likely cause of specific migration is the release of chemotactic gradients from the tumors, which may enable MSCs to home to, and modulate, the tumor microenvironment [5,6]. Owing to these properties and their ability to modulate the activity of immune cells, MSCs could function as cellular delivery vehicles for anti-tumor agents [7-9].

MSCs were first identified in the 1960s in the stromal compartment of bone marrow [10,11], and since then, they have been isolated from a wide variety of adult [12-20] and fetal (both first and second trimester) tissues, including blood, liver, bone marrow, placenta, and umbilical cord [21-25], using similar techniques [26]. The best-characterized source for adult human stem cells is bone marrow, and both bone marrow-derived human MSCs (BM-hMSCs) and adipose-derived human MSCs (hASCs) have become attractive candidates because these tissues are rich sources of MSCs and are easy to collect. The other tissue-derived MSCs share a number of important characteristics with BM-hMSCs, including expression of cell surface marker, ability to adhere to plastic, and capacity to differentiate into cells of mesenchymal lineage under appropriate conditions [27]. Despite extensive investigations, the effect of unmodified MSCs on tumor progression remains unclear. Many studies have shown that MSCs promote tumor progression and metastasis, whereas others have reported that MSCs suppress tumor growth [28]. The contradictions in these findings may be attributable to the variability and heterogeneity in adult stem cells from different sources, or to differences in isolation methods and *in vitro* culture conditions. Further development of an efficient and safe cell-based therapy will require the *in vivo* tracking of engrafted MSCs to ensure that they reach their destination. *In vivo* imaging techniques provide a continuum observation rather than a single snapshot of conventional post-mortem histological analyses.

The aim of our work was to investigate the efficacy and efficiency of five different MSC lineages, in order to assess their adequacy for use as cell-based anti-tumor therapies. Our study shows the crucial importance of understanding the interaction between MSCs and tumor cells, and provides both information and a methodological approach, which could be used to develop safer and more accurately targeted therapeutic applications. The pluripotency expression pattern of MSCs was studied and compared with that obtained in human induced pluripotent stem cells (hiPSCs). Furthermore, the effects exerted on migration-related gene expression in tumors obtained from animals after 24 days of systemic MSC injection were also analyzed.

## Methods

### Cell cultures

A human cervical cancer cell line (HeLa; Cancer Research UK Cell Services, London Research Institute, Clare Hall Laboratories, Herts, UK) and human PN3 fibroblasts (kindly supplied by Dr Liu (Imperial College, London, UK)) were used. Cells were cultured in DMEM containing 10% FBS and antibiotics (Lonza, Verviers, Belgium), at 37°C in 5% CO_2_.

All MSC media were supplemented with 10% FBS and antibiotics. BM-hMSCs were obtained from Lonza and maintained in DMEM low glucose (1.0 g/l) and hypoxic conditions (3% O_2_). hASCs were obtained from Invitrogen (UK) and cultured in MesenPro RS Basal Medium and MesenPro RS Growth Supplement (Gibco, Paisley, UK). Human epithelial endometrium-derived stem cells or hEESCs (also known as endometrial epithelial stem cell lines; ICEp) and human stroma endometrium-derived stem cells or hESSCs (also known as endometrial stromal stem cell lines; ICEs) were supplied by Dr Carlos Simon from IVI (Valencia, Spain) [12,13]. Cells were maintained in DMEM F-12 under hypoxic conditions (3% O_2_) and dishes were pre-treated with 0.1% gelatin solution (Sigma-Aldrich Chemie GmBh, Munich, Germany). Human amniotic membrane mesenchymal stem cells or hAMCs were obtained from Cellular Engineering Technologies (CET), (Coralville, IA, USA) and were maintained in DMEM high glucose (4.5 g/l) and 10 ng/ml basic human fibroblast growth factor (hFGFb;Gibco). Cells were used between passages 5 to 8.

The hiPSCs (human IPSC line 2 F8) were kindly supplied by Dr Austin Smith (University of Cambrige, UK) and cultured in knockout DMEM (Gibco), 15% knockout serum (Gibco), 1× NEAA (Lonza), 0.1 mmol/l β-mercaptoethanol (Sigma-Aldrich), 10 ng/ml hFGFb, and antibiotics at 37°C in 5% CO_2_. Cells were seeded on a PN3 feeder cell monolayer inactivated with mitomycin C (Sigma-Aldrich).

### Flow-cytometry analysis

Characterization of MSCs was verified by flow cytometry. The negative surface markers used were CD45, CD34, and HLA-DR, and the positive ones were CD90, CD73, and CD105, plus CD9 and CD13 (the last two markers refers only to hEESCs and hESSCs, respectively). The antibodies used were CD13 FITC-conjugated (Immunostep, Salamanca, Spain), CD34 Percp/Cy5.5-conjugated (Becton Dickinson Co., Madrid, Spain), CD9 PE-conjugated (Millipore Corp., Billerica, MA, USA), CD45 PerCP/Cy5.5-conjugated (Becton Dickinson), CD73 PE-conjugated (BD), CD90 PE-conjugated (Becton Dickinson), CD105 FITC-conjugated (R&D Systems Inc., Minneapolis, MN, USA), and HLA-DR APC-conjugated (Immunostep). Briefly, cells were incubated in PBS supplemented with 2% FBS and specific antibodies at 4°C for 30 minutes. Then, cells were washed and fixed in 1% paraformaldehyde (Sigma-Aldrich) before FACS analysis (FACSAria system; Becton Dickinson).

### *In vitro* MSC differentiation

For adipogenesis, MSCs were kept for 21 days in 1× basal medium (STEMPRO® Adipocyte Differentiation Basal Medium; Invitrogen Corp., Carlsbad, CA, USA), 1× supplement (STEMPRO® Adipogenesis Supplement; Invitrogen) and antibiotics. When differentiation was finished, cells were stained with Oil Red O solution (Sigma-Aldrich). For osteogenesis, MSCs were maintained for two weeks in DMEM medium containing 10% FBS, 50 μg/ml ascorbic acid, 100 nmol/l dexamethasone, and 10 mmol/l β-glycerophosphate disodium salt hydrate (Sigma-Aldrich) and antibiotics. Osteocyte formation was evaluated by staining with Alizarin Red S (Sigma-Aldrich). Images were visualized under a microscope (AE31; Motic Group Co. Ltd, Causeway Bay, Hong Kong) equipped with a camera (2500 Moticam; Motic Group) and Motic Imaging Plus 2 software (version 0.23).

### Adenoviral vectors and infections

The hNIS gene is endogenously expressed mainly in the thyroid and stomach, and is responsible for iodide concentration. In cells expressing hNIS, gamma ray-emitting radioisotopes such as ^99m^Tc are accumulated, and can be imaged by SPECT-CT, and thus the hNIS gene can be used as a reporter gene [29]. The adenoviral vector AdhNIS (also known as Ad10) used in this work was based on adenovirus serotype 5, and the hNIS gene is driven by the immediate-early cytomegalovirus promoter. AdhNIS was constructed and amplified as previously described [30]. The amount of infective adenoviral vector per cell (pFUs/cells) in culture media was expressed as multiplicity of infection (MOI). Previously, adenoviral infection efficiency was determined using adenoviral vector AdGFP testing at 100, 250, 500, and 1000 MOI, as in our previous study [31] (data not shown). For the adenoviral infection with AdhNIS, viruses were diluted in serum-free culture media to 500 MOI, added to cells, and incubated at 37°C for 1 h. The complete medium was then added and cells were maintained for 24 h until used in the *in vivo* experiments.

### Ethics approval

All procedures were carried out under a project license approved by the Ethics Committee for Animal Experiments from the University of Zaragoza (Spain). The care and use of animals was performed in accordance with the Spanish Policy for Animal Protection RD1201/05.

### Experimental *in vivo* design

Female BALB/c nu/nu mice 6–8 weeks old (Harlan UK Ltd (Bicester, Oxfordshire, UK) and Harlan Interfauna Iberica (Barcelona, Spain)) received subcutaneous (SC) injections of 2 × 10^6^ HeLa cells suspended in 200 μl PBS for the generation of subcutaneous xenograft tumors. When these tumors reached 50 mm^3^ in size, mice were randomly divided into different groups, and intravenous injections of MSC were performed. For MRI experiments, animals were separated into six groups (n = 4/group). Group 1 (BM-hMSCs injected); group 2 (hASCs injected); group 3 (hAMCs injected); group 4 (hESSCs injected); group 5 (hEESCs injected); and group 6 (control; PBS injected). For SPECT-CT experiments, animals were separated into seven groups (n = 4/group), with groups 1 to 5 as above, and groups 6 and 7 being injected with hiPSCs or PBS (control), respectively.

### Iron-oxide labeling and cell-viability assay

MSCs were magnetically labeled with superparamagnetic iron oxide (SPIO; Endorem, Guerbet, France), as previously described [32]. SPIO is an oxide nanoparticle solution with a total iron content of 11.2 mg Fe/ml. Labeling with SPIO acts by reducing the transverse relaxation time on T2-weighted MRI scans. Cells were incubated with the labeling medium containing 100 μg/ml iron for 24 h. After labeling, cells were washed to remove residual contrast agent.

Viability of the iron oxide-labeled MSCs was evaluated by performing a long-term (10 days) *in vitro* exclusion test with Trypan blue (Sigma-Aldrich).

### MRI

Animals bearing the tumor xenograft were separated into six groups (n = 4/group) when the tumors reached 50 mm^3^. MSCs were labeled with SPIO as described above. Groups 1 to 5 received an intravenous injection of 10^6^ SPIO-labeled MSCs, while the control group (group 6) received intravenous injection of PBS. Scans were performed at 3, 10, 17, and 24 days after injection. The MRI experiments were performed (Pharmascan system; Bruker Medical GmBH, Germany, http://www.bruker-biospin.com/pharmascan.html) using a 7.0-T horizontal-bore superconducting magnet, equipped with a ^1^H selective surface coil and a Bruker gradient insert with 90 mm inner diameter (maximum intensity 360 mT/m). All data were acquired using Paravision software (Bruker). Anesthesia was initiated using oxygen (1 l/min) containing 4% isofluorane, and maintained during the experiment with 1 to 1.5% isofluorane in O_2_. T2-weighted spin-echo anatomical images were acquired by rapid acquisition with relaxation enhancement (RARE) sequence in axial (12 slices) and sagittal (8 slices) orientations and the following parameters: TR 3000 ms, TE 60 ms, RARE factor 8, average 3, FOV 30 × 30 mm, acquisition matrix 256 × 256, corresponding to an in-plane resolution of 117 × 117 μm^2^ and slice thickness of 1.00 mm. Tumors were measured every 2 days and tumor volume was calculated using the formula:

tumor volume = 1/2 L × S^2^, where L is long side and S is short side.

### Prussian blue staining and histological analysis

At day 24, the animals in the *in vivo* MRI experiments were euthanized. Tumors and tissues were obtained, fixed in formalin, and embedded in paraffin wax. Sections of 4 μm were obtained from the blocks for staining with haematoxylin and eosin (Sigma-Aldrich). Staining with Prussian blue (Sigma-Aldrich) was used to detect labeled iron particles in the cells, in accordance with the manufacturer’s instructions. The number of blue-stained (positive) cells per high-power field (HPF) was calculated by counting the cells in at least five HPFs per section, with a minimum of five sections per sample examined.

### SPECT-CT imaging

Animals bearing tumor xenograft were separated into seven groups (n = 4/group) when the tumors reached 50 mm^3^. MSCs were infected with AdhNIS as described above. Groups 1 to 6 received intravenous injection of 10^6^ hNIS-labeled MSCs and the control group (group 7) received intravenous injection of PBS. At 3, 10, 17, and 24 days post-injection, all groups received an intravenous dose of 18.5 MBq of ^99m^Tc. Anesthesia was initiated by inhaled oxygen (1 l/min) containing 4% isofluorane, and maintained during the experiment with 1 to 1.5% isofluorane in O_2_. The mice were scanned using a nano-SPECT-CT scanner for small animals (Bioscan, Paris, France). A tomogram was taken, and the limits of the scan were determined. A CT and a SPECT whole-body scan were performed, with a time of 100 seconds per acquisition. The images were reconstructed with the MEDISO software (Medical Imaging Systems, Budapest, Hungary); fusion of SPECT and CT images was carried out using PMOD software (Biomedical Imagen Quantification, Basel, Switzerland). Tumors were measured every two days and tumor volume was calculated using the formula for tumor volume given above.

Quantification of radioisotope (^99m^Tc) accumulation was carried out using InVivoScope software (Medical Imaging Systems). Fused SPECT/CT images were used to draw the voxel-guided specific volume of interest (VOI) to accurately quantify the total activity associated with the whole tumor volume. Quantification of ^99m^Tc accumulation was expressed as ratio of tumor uptake to muscle uptake.

### Reverse transcription-PCR

Total RNA was isolated from tumors from the *in vivo* SPECT-CT experiment using a commercial kit (NucleoSpin RNA II Kit; Macherey-Nagel GmBH, Dueren, Germany), in accordance with the manufacturer’s instructions. Total RNA was reverse-transcribed to cDNA (SuperScript II Reverse transcriptase; Invitrogen). Control reactions were performed by omitting the reverse transcriptase. Reverse transcription (RT)-PCR was carried out using hNIS-specific and GAPDH-specific primers (Table 1). PCR was performed using an automated system (Applied Biosystems 2720 Thermal Cycle System; Life Technologies, Glasgow, UK). cDNA products were separated by electrophoresis.

**Table 1:** Primers used for PCR

**Primer**	**Direction**	**Sequence 5′→3′**
hNIS	Sense	CCCATCGATGGAGGCCGTGGAGACCGG
	Anti-sense	CCCATCGATGTCAGAGGTTTGTCTCCTGC
GADPH	Sense	CCCATCGATGTCAGAGGTTTGTCTCCTGC
	Anti-sense	GGTCATGAGTCCTTCCACGAT

*GADPH* glyceraldehyde-3-phosphate dehydrogenase, *hNIS* human sodium iodine symporter.

### Real-time quantitative PCR

#### Pluripotency-related genes

Real-time quantitative PCR (qPCR) was performed to analyze expression of NANOG, SOX2, OCT4, KLF4, and REX1. To detect gene expression, MSCs and hiPSCs were lysed directly in the culture dishes, then total RNA was extracted and reverse transcription to cDNA was carried out as described above. To test the quality of the cDNA, mRNA of the housekeeping GADPH was amplified. RNA from hiPSCs was used as pluripotency control. Reactions were performed using a master mix (Taqman Universal Master Mix II; Invitrogen) and a PCR system (Applied Biosystems 7900HT Fast-Real Time PCR System; Life Technologies) with probes (Applied Biosystems. Foster City, CA, USA) (Table 2). Gene expression levels were normalized against 18S rRNA. The ratio of the relative expression for each gene to 18S was calculated by the 2^-ΔΔCq^ formula. The hiPSC data were used as a calibrator for the results presented (2^-ΔΔCq^ = 100%).

**Table 2:** TaqMan® gene expression assays used to amplify the pluripotency-related genes

**Gene**	**Assay ID**	**Amplicon size, nucleotides**	**Dye**
NANOG	Hs02387400_g1	109	FAM
SOX2	Hs01053049_s1	91	FAM
OCT4	Hs00742896_s1	65	FAM
KLF4	Hs00358836_m1	110	FAM
REX1	Hs00358836_m1	102	FAM
18S	4352930E	187	FAM/MGB

#### Migration-related genes

qPCR was performed to analyze expression of CXCL12 (SDF-1), CXCR4, MMP-2, CCL2 (MCP-1), and CCL5 (RANTES) (Sigma-Aldrich) (Table 3). To detect and quantify gene expression, total RNA from tumors consisting only of HeLa cells (control group) and tumors consisting of HeLa cells with MSCs or hiPSCs injected intravenously were isolated and then reverse-transcribed to cDNA. Reactions were performed using SYBR Green (Power SYBR Green PCR Master Mix; Life Technologies) and a real-time PCR system (Applied Biosystems 7500; Life Technologies). Reactions were performed in duplicate from three different dilutions, and threshold cycle values were normalized to the housekeeping gene 18S. The specificity of the products was determined by melting curve analysis and gel electrophoresis. The ratio of the fold difference expression for each gene to 18S was calculated using the ^-ΔΔ^Cq formula. Gene expression levels were normalized against 18S rRNA and the expression obtained from the control group was used as a calibrator (^-ΔΔ^Cq = 0).

**Table 3:** Primers used to amplify the migration-related genes

**Primer**	**GenBank accession number**	**Direction**	**Sequence (5′ → 3′)**
CXCR4	NM_003467	Sense	TGGCCGACCTCCTCTTTGT
		Anti-sense	AGTTTGCCACGGCATCAACT
SDF-1 (CXCL12)	NM_000609	Sense	CCAACGTCAAGCATCTCAAAATT
		Anti-sense	AGCCGGGCTCAATCTGAAG
RANTES (CCL5)	NM_002985	Sense	TGCCCACATCAAGGAGTATTTCTA
		Anti-sense	GCACACACTTGGCGGTTCT
MCP-1 (CCL2)	NM_002990	Sense	GCGTGGTGTTGCTAACCTTCA
		Anti-sense	GGCTCTTCATTGGCTCAGCTT
MMP-2	NM_004530	Sense	TTTTGATGACGATGAGCTATGGA
		Anti-sense	CCATCGGCGTTCCCATACT
18S	NR_003286	Sense	CGGCTACCACATCCAAGGAA
		Anti-sense	GCTGGAATTACCGCGGCT

### Statistical analysis

Results are reported as mean ± SEM. Statistical evaluation of data was carried out using the SPSS Statistics software package (version 17.0; IBM SPSS, Chicago, IL, USA). Normal distribution of the variables was analyzed by means of the Kolmogorov-Smirnov test followed by the Tukey HSD test, except for KLF4 and REX1 expression in the pluripotency qPCR assays, whose distribution was non-parametric and so analysis was performed using the Kruskal-Wallis test. *P*<0.05 was considered significant.

## Results

To confirm whether labeled MSCs injected systemically into animals are able to migrate, proliferate, and engraft into the microenvironment of tumors, two non-invasive imaging techniques were performed in the present study: 1) SPECT-CT using hNIS as reporter gene, and 2) MRI labeled with iron nanoparticles (SPIO).

**Figure 1:**
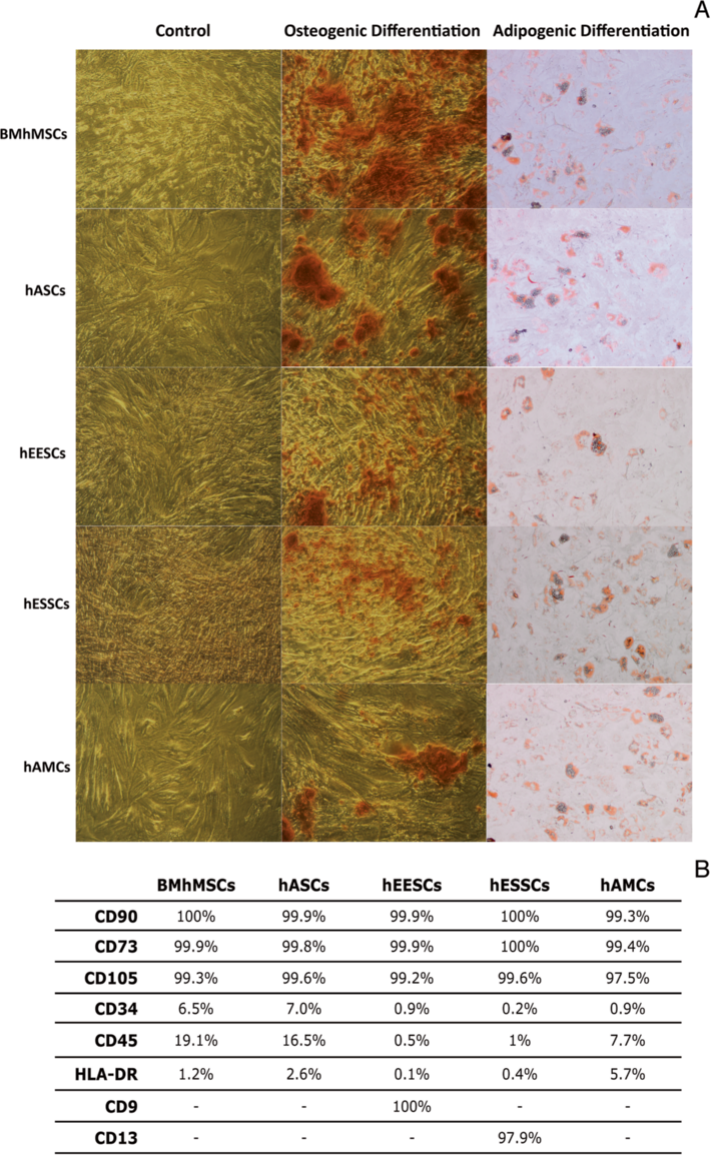
**Characterization of MSCs. (A)** Multi-lineage differentiation potential *in vitro* to give rise to osteoblasts and adipocytes. Morphologic changes and expression of specific markers for each tissue are shown. **(B)** Specific surface markers expression (%) detected by flow cytometry.

Before performing specific studies, it was tested whether these cells gave rise to adipocytes and osteoblasts when they were placed in specific differentiating conditions to show their multi-lineage differentiation potential (Figure 1A). In addition, it was verified that cells were negative (≤19%) for CD45, CD34, and HLA-DR surface markers, and positive (≥97%) for CD90, CD73, and CD105, as well as CD9 and CD13 (the latter two specific for hEESCs and hESSCs, respectively) (Figure 1B).

### Cell-viability assay of SPIO-labeled MSCs

Before performing MRI scans, viability of SPIO-labeled MSCs was determined by Trypan blue exclusion assay. Cell-viability values, compared the control group (100%), with 99.23 ± 3.18% in the BM-hMSC group, 101.53 ± 5.6% in the hASC group, 96.4 ± 1.75% in the hEESC group, 100.28 ± 0.97% in the hESSC group, and 98.05 ± 6.15% in the hAMC group, thus revealing that labeling with SPIO did not significantly affect cell viability and proliferation.

### *In vivo* tropism of MSCs to tumors, detected by non-invasive imaging techniques

**Figure 2:**
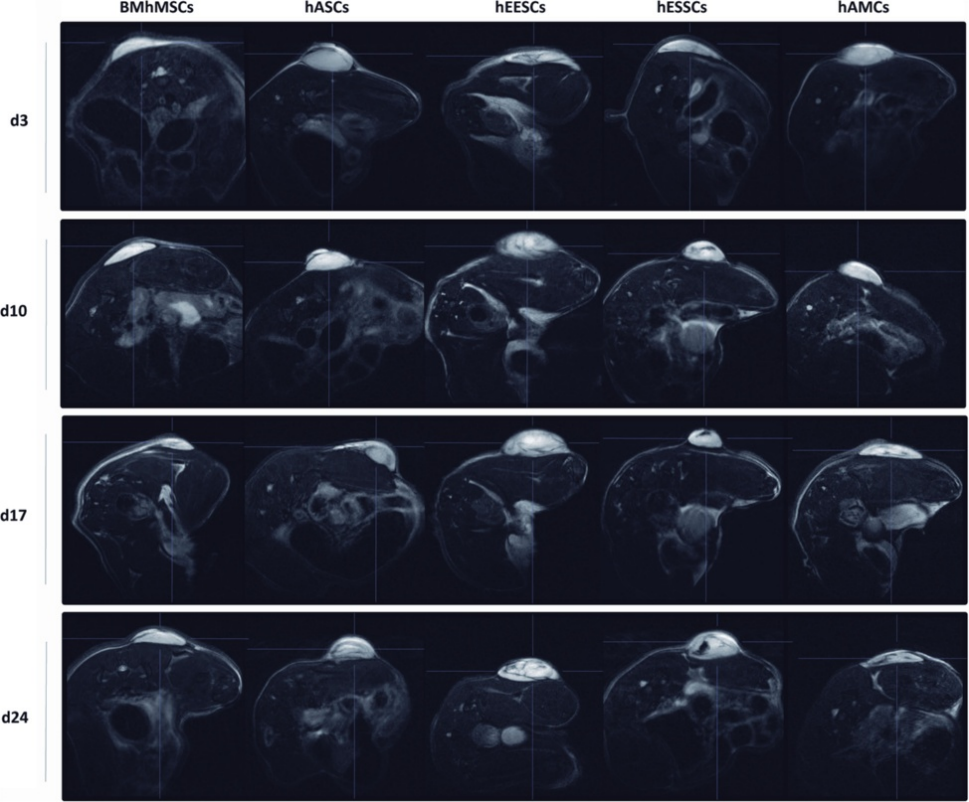
***In vivo *xenograft imaging of mice using a magnetic resonance labeling (MRI) scanner.** Scans were performed at 3, 10, 17, and 24 days after intravenous injection of super-paramagnetic iron oxide (SPIO)-labeled mesenchymal stem cells (MSCs). Tumors were visible as lighter areas in transverse sections. The recruitment of SPIO-labeled MSCs to tumors resulted in decrease of signal intensity (SI) and the visualization of darker areas in tumor sites. The same animals are represented over the entire period.

**Figure 3:**
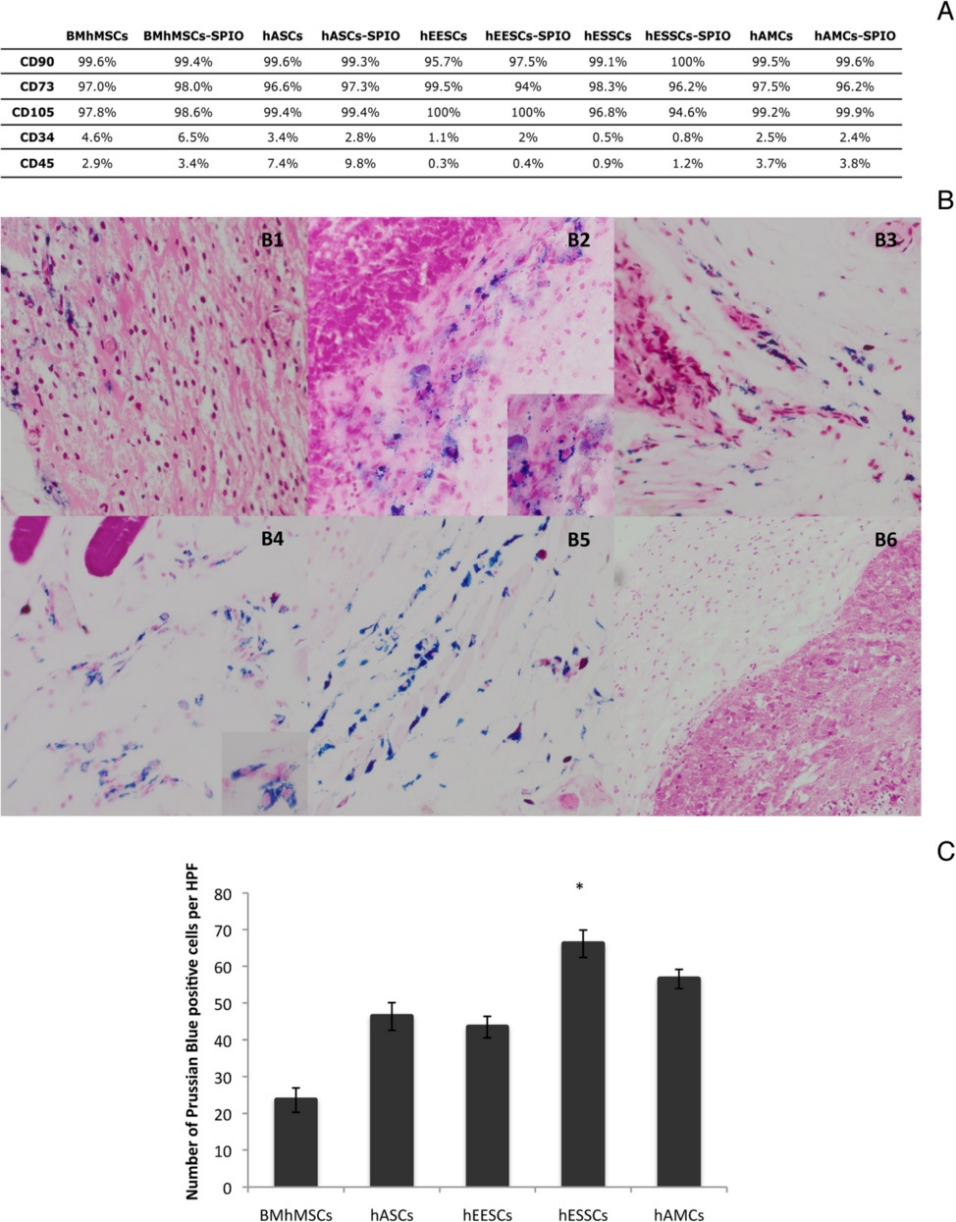
**Super-paramagnetic iron oxide (SPIO) labeling analysis. (A)** Phenotypic analysis after SPIO labeling. Prussian blue staining in tumor sections obtained from animals scanned by MRI at day 24. **(B)** Images show intense blue clusters in tumor sections. **(B1)** SPIO-labeled bone marrow-derived human mesenchymal stem cells (BM-hMSCs); **(B2)** SPIO-labeled human adipose-derived stem cell (hASCs); **(B3)** SPIO-labeled human epithelial endometrium-derived stem cell (hEESCs); **(B4)** SPIO-labeled human stromal endometrium-derived stem cells (hESSCs); **(B5)** SPIO-labeled human amniotic membrane mesenchymal stem cells (hAMCs; **(B6)** control cells. Original magnification: (B1-B5) ×20; (B6) ×10. **(C)** number of Prussian blue-stained positive cells per high-power field (HPF) in tumor sections.

The ability of MSCs to migrate *in vivo* towards tumor sites was first determined through MRI (Figure 2). The study of expression of surface markers by flow cytometry did not bring about changes in MSCs phenotype after SPIO labeling (Figure 3A). The recruitment of SPIO-labeled MSCs to tumors resulted in a decrease in signal intensity (SI) and visualization of darker areas in tumor sites. Tumors were visible as lighter areas. The changes in SI were detected by T2-weighted images. Although no decrease in SI was detected in control animals (data not shown), changes in SI were seen in the different MSCs injected from the first determination at day 3. Images of hASCs, hEESCs, and hESSCs at day 3 showed a higher SI decrease than hAMCs and BM-hMSCs. These differences were more pronounced from day 10, thus highlighting the differences in the ability of certain cells to migrate towards tumor areas. In all groups, SI decrease was observed first around the tumor sites and then inside the tumors, and was much more significant in hEESCs and hESSCs. To confirm these data, Prussian blue staining was used to detect SPIO-labeled MSCs in tumor sections obtained from animals scanned at day 24. Intense blue clusters were seen in tumor sections (Figure 3B). Higher-magnification images show the intra-cytoplasmic localization of the iron particles and the absence of extracellular iron. The number of Prussian blue-stained positive cells per HPF was calculated in tumor sections (Figure 3C). There were significantly more Prussian blue-stained positive cells in tumor sections of hESSCs (66.1 ± 3.72) compared with BM-hMSCs (23.6 ± 3.30).

**Figure 4:**
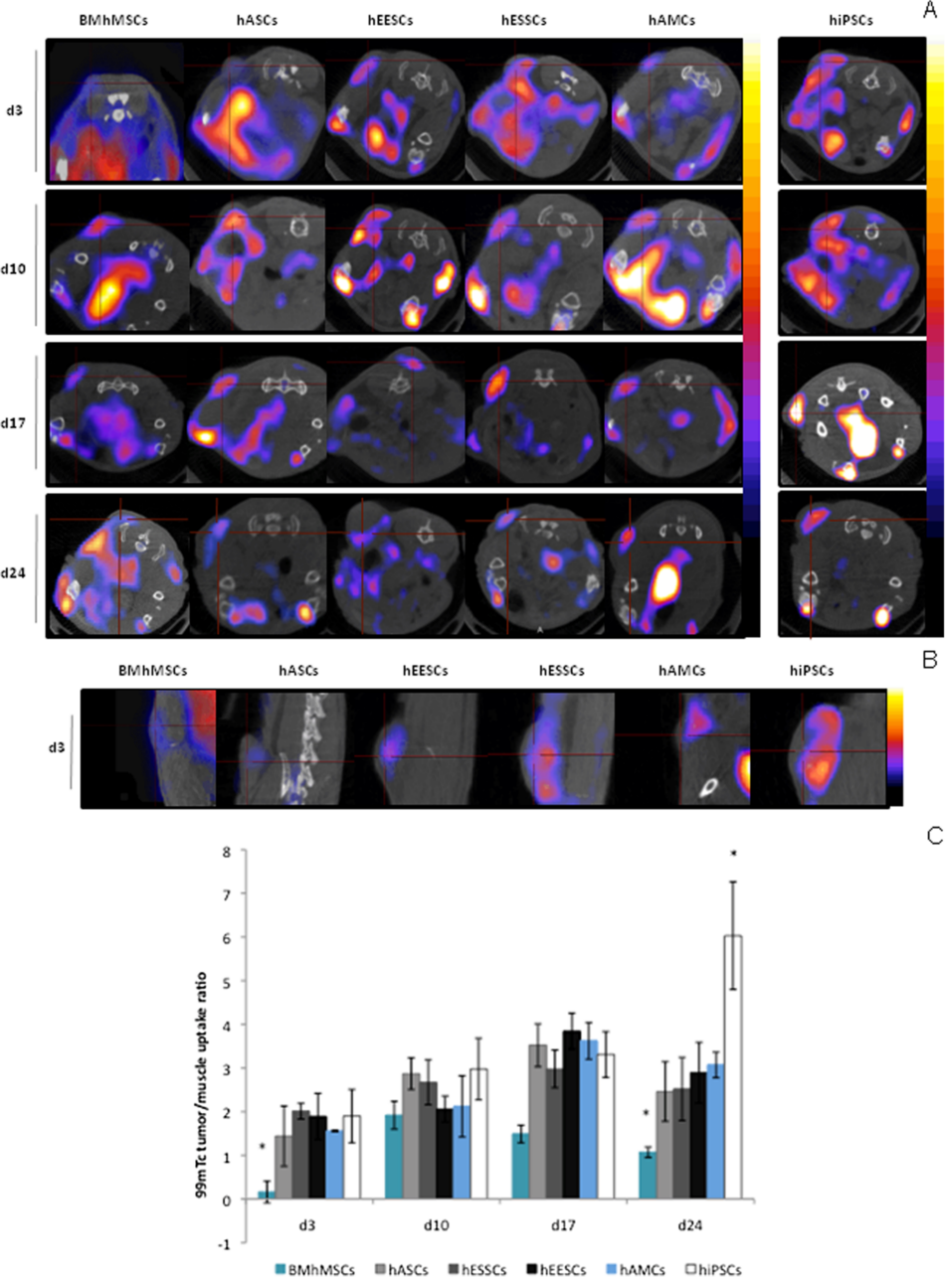
***In vivo *xenograft imaging of mice using a nano-single-photon emission computed tomography/X-ray computed tomography (nano-SPECT-CT) scanner.** Scans were performed at 3, 10, 17, and 24 days after intravenous injection of human sodium iodine symporter (hNIS)-labeled mesenchymal stem cells (MSCs) and human induced pluripotent stem cells (hiPSCs). An intravenous dose of 18.5 MBq of ^99m^Tc was administered before the acquisitions. The same animals are represented over the entire period. hNIS expression is indicated by red crosses in transverse sections. Scans were performed after intravenous injection of hNIS-labeled MSCs or hiPSCs. **(A)** Images at days 3, 10, 17 and 24; **(B)** images at day 3; **(C)**: ^99m^Tc tumor/muscle uptake ratio by hNIS-labeled MSCs or hiPSCs from days 3 to 24.

A second *in vivo* imaging technique, SPECT-CT, was performed. Selected tumor transverse sections of SPECT-CT scans are shown in Figure 4A. The images obtained by SPECT-CT display a color spectrum, with the weakest signal being blue and the strongest red. In whole-body scans of control mice, an endogenous hNIS expression signal was present in the thyroid gland, salivary glands, stomach, and bladder. Accumulation of ^99m^Tc, reflecting hNIS expression, was detected at tumor sites in animals that received the hNIS-labeled MSC injection. No signal was detected in tumor sites of the control animals (data not shown). Because MRI assays indicated differences in signal levels at first determinations, hiPSCs were included in the SPECT-CT assays to study whether pluripotency status can affect migration ability.

hMSCs did not exert any signal at day 3, and their highest signal was found at day 10 (1.92 ± 0.72), after which it decreased until day 24 (1.07 ± 0.12). In hiPSCs and in the other hMSCs assayed, various levels of signal were detected from day 3, with hASCs presenting the lowest signal (1.44 ± 0.69) and hESSCs the highest (2.01 ± 0.18). Figure 4B shows the differences at day 3. The detected signals increased for the following determinations, with MSCs reaching their maximum level at day 17, when hESSCs also had their highest values (3.84 ± 0.41), whereas the highest signal for hiPSCs (6.73 ± 1.23) occurred at day 24. For MSCs, the detected signals decreased after 24 days, with the highest values being for hAMCs (3.07 ± 0.29) and hESSCs (2.89 ± 0.69), and the lowest for hASCs (2.46 ± 0.68) and hEESCs (2.52 ± 0.72). The tumor:muscle uptake ratio showed the differences between the migration ability of MSCs and hiPSCs (Figure 4C).

**Figure 5:**
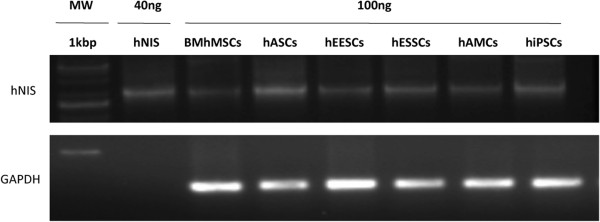
**Detection of human sodium iodine symporter (hNIS) expression by reverse transcription (RT)-PCR.** RNA was extracted from tumors of animals scanned by single-photon emission computed tomography/X-ray computed tomography (SPECT-CT) at day 24.

To confirm MSC and hiPSC engraftment, hNIS expression was performed by RT-PCR (Figure 5). At day 24, the animals were euthanized, and RNA was extracted from tumors as described above. hNIS expression was detected in all groups with differences of intensity. These differences may be due to the differences seen in the distribution of MSCs and hiPSCs in tumors and also to episomal hNIS expression mediated by adenovirus. Findings suggest that MSCs and hiPSCs have the ability to migrate to and engraft into tumor sites. In SPIO-labeled MSCs and in hNIS-labeled MSCs and hiPSCs, the signal was maintained for up to 24 days after injection, depending on the cell type. BM-hMSCs showed the lowest migration ability, being detected in tumor sites at day 10, whereas hiPSCs showed the highest capacity, with increased signals until day 24. The other MSC types were detected from the first determination (day 3) and the signal was stronger until the final determination (day 24), thus revealing that more cells can reach tumor areas and remain there for longer periods.

### Pluripotency analysis

**Figure 6:**
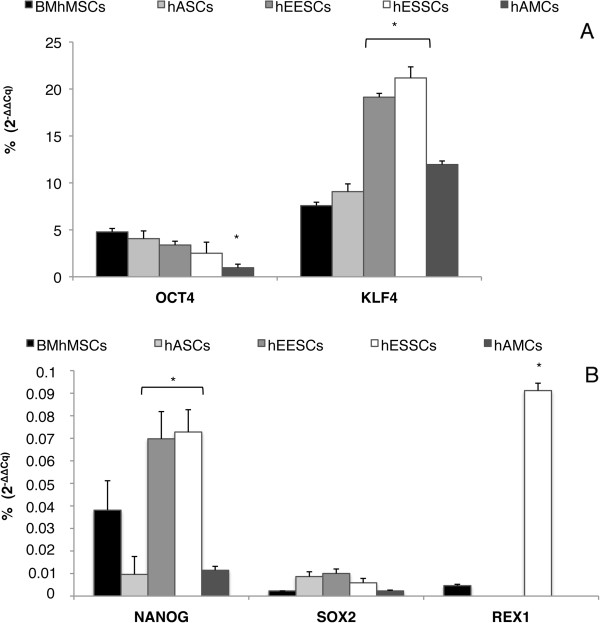
**Pluripotency-related gene expression by quantitative (q)PCR.** Gene expression levels were normalized against 18S rRNA. The ratio of the relative expression for each gene to 18S was calculated by using the 2^-ΔΔCq^ formula. hiPSC data were used as a calibrator for the results presented (100%). **(A)** OCT4 and KLF4; **(B)** NANOG, SOX2 and REX1.

To investigate whether pluripotency may be involved in the migration ability of MSCs, analysis of the expression of pluripotency-related genes was performed (Figure 6). qPCR analysis showed that the expression of the genes studied was significantly lower in MSCs compared with the control sample, hiPSCs (taken as 100%). Moreover, differences were found in the expression levels between the MSCs. NANOG, and KLF4 expression was higher in hEESCs and hESSCs than in the other MSCs. There were no significant differences in SOX2 expression between the different MSCs, and OCT4 levels were very similar for MSCs as well, except for hAMCs in which expression was significantly lower (0.96%). REX1 was expressed only in hESSCs and BM-hMSCs (<0.01%). These results clearly show that although all cell types in the study may be considered MSCs, they exert a very different pluripotency pattern, and are also different from hiPSCs, which may be related to the differences in their engraftment capacity in addition to their effects on tumors.

### Effects on xenograft tumor growth

**Figure 7:**
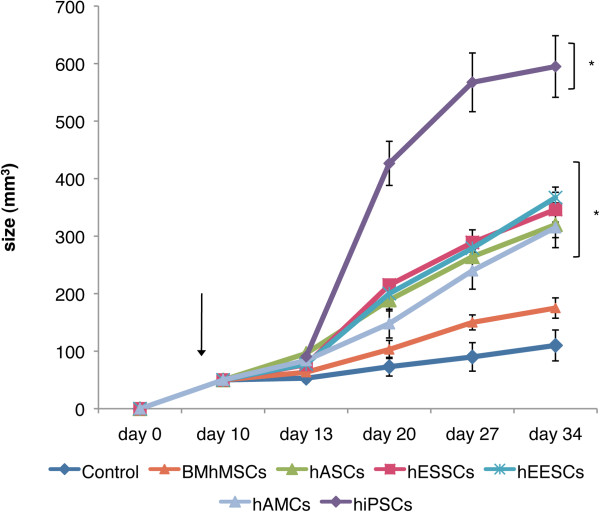
**Effect on xenograft tumor growth.** When tumors reached a size of 50 mm^3^, the animals received an intravenous injection of mesenchymal stem cells (MSCs; arrow). The size of the tumors was measured until the end of the experiments (34 days after the HeLa injection and 24 days after the MSC injection).

When tumors reached a size of 50 mm^3^, approximately 10 days after HeLa injection, the animals received intravenous injection of MSCs or hiPSCs (Figure 7). Tumor size was measured until the end of the experiments (34 days after HeLa injection and 24 days after MSC or hiPSC injection). The growth of the tumors was clearly different depending on the type of MSCs or hiPSCs injected. Until day 13, the tumors of all the groups showed a similar growth pattern. However, differences between groups became clear at day 20, and were maintained until day 34. At this point, control tumors consisting of HeLa cells had an average size of 110 ± 26.8 mm^3^, whereas tumors composed of HeLa cells and MSCs had faster growth. Tumors resulting from the hEESC, hESSC, hASC, and hAMC injections reached a similar average size between 346 and 315 mm^3^, significantly larger than the control tumors except for those resulting from BM-hMSC injections (174.9 ± 17.7 mm^3^). Tumors composed of HeLa cells and hiPSCs had the fastest growth rates and reached an average size of 594.8 mm^3^, significantly larger than those in the other groups. The significantly higher growth of HeLa cells and MSC or hiPSC tumors compared with the control group indicates migration and engraftment of these cells into tumors.

### Migration analysis

**Figure 8:**
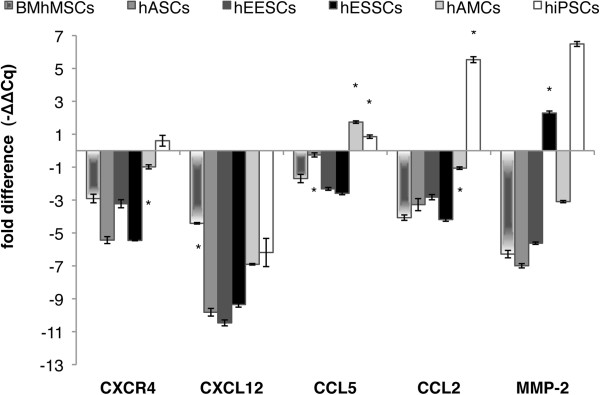
**Migration-related gene expression by quantitative (PCR).** CXCR4, CXCL12, CCL5, CCL2, and MMP-2 expression in tumors (control group) was compared with expression in tumors consisting of HeLa cells and mesenchymal stem cells (MSCs) or of HeLa cells and human induced pluripotent stem cells (hiPSCs) (analyzed 24 days after stem cell injection). The ratio of the fold difference expression for each gene to 18S was calculated by using the ^-ΔΔ^Cq formula. Expression obtained from the control group was used as a calibrator.

To compare the expression of migration-related genes in tumors consisting only of HeLa cells (control group) and tumors consisting of HeLa cells and MSCs or hiPSCs, mRNAs encoding CXCR4, CXCL12, CCL5, CCL2, and MMP-2 were quantified by qPCR (Figure 8).

In general, expression levels of migration-related genes in tumors obtained from HeLa cells and MSC or hiPSC injection were significantly lower than those of control samples. CXCL12 expression was significantly decreased in all groups, with the smallest decrease seen in BM-hMSCs (−4.4 ± 0.04-times), and the largest in hASCs, hEESCs, and hESSCs (between −9-fold and −11-fold). For CCL5, expression levels were significantly decreased in tumors obtained from BM-hMSCs (−1.69 ± 0.25-fold), hEESCs (−2.31 ± 0.08-fold), and hESSCs (−2.58 ± 0.08-fold), but were significantly increased in hAMCs (1.73 ± 0.07-fold) and hiPSCs (0.85 ± 0.1-fold). In the hASC group, the expression level was similar to that of the control samples (−0.25 ± 0.12-fold). CCL2 and CXCR4 expression levels were significantly decreased in tumors resulting from MSC injection, with the hAMC group showing the lowest decrease (−1.05 ± 0.07-fold and −0.98 ± 0.12-fold, respectively), whereas the hiPSC group had significantly increased expression levels (5.53 ± 0.18-fold and 0.60 ± 0.33-fold, respectively). Finally, MMP-2 expression was significantly decreased in tumors composed of HeLa cells and BM-hMSCs (−6.28 ± 0.22-fold), hASCs (−6.99 ± 0.12-fold), hEESCs (−5.62 ± 0.13-fold), and hAMCs (−3.09 ± 0.06-fold) but was significantly increased in hESSCs (2.28 ± 0.07-fold) and hiPSCs (6.48 ± 0.14-fold). These findings highlight, once again, the differences between MSCs and hiPSCs. These differences may be responsible for the different migration patterns and probably play a role in the effects on the microenvironment in tumors.

## Discussion

MSCs have been promoted as an attractive option for cellular delivery vehicles to carry anti-tumor agents, owing to their ability to home into tumor sites and secrete cytokines [28,33,34]. Previous studies have shown that systemic delivery of MSCs does not result in engraftment into healthy organs, but that they do migrate in various *in vivo* tumor models, although this mechanism has not yet been fully elucidated [35-42]. Additionally, MSCs can also counteract inflammation by suppressing host immune responses [28] and by secreting anti-inflammatory cytokines [43].

The ability of MSCs and hiPSCs to migrate into tumor sites after systemic injection into mice is confirmed in the present study. HeLa-based subcutaneous xenograft tumors showed extensive MSC engraftment, and BM-hMSC migration was significantly lower and slower than that obtained by the other MSCs and hiPSCs. All cell types were detected by MRI in a similar way to recent results obtained with MSCs by other groups [43]. Signals were obtained from the first (day 3) until the final (day 24) imaging determination, except in BM-hMSCs, which highlights the capacity of these cells to persist in tumor sites. These results and the differences between BM-hMSCs and the other cell types were confirmed by iron-particle staining and the detection of hNIS in tumor samples. Using SPECT-CT images and hNIS expression, another study has shown BM-hMSC migration towards a breast-cancer model [44]. In contrast to our observations, hNIS expression in that study was detected in tumors from day 3 after MSC injection, which may be attributed to the differences between donors, tumor models, and the size of the pre-established tumor.

To clarify whether pluripotency plays a role in MSC migration to tumors, a pluripotency marker panel was analyzed. These studies revealed diminished expression of SOX2, NANOG, OCT4, KLF4, and REX1 compared with the control sample (hiPSCs). hEESCs and hESSCs, which displayed the highest signals in the imaging techniques, also had significant expression of KLF4, NANOG, and REX1 with respect to the other MSCs, although it was much lower than that shown by hiPSCs. These differences in pluripotency patterns may influence the differences in migration and engraftment of MSCs and hiPSCs.

Tumor growth confirmed the ability of MSCs to migrate into tumors, and the significant differences between BM-hMSCs, the other MSCs, and hiPSCs. In a previous PC3 prostate xenograft model [45], intra-tumoral injection of hASCs also induced larger tumors compared with the control group, although the differences between control and hASC-induced tumors were smaller at the final time point (<2-fold vs. 3-fold in ours). These differences may be attributed to the different tumor model used and the different procedures for hASC injection. However, there are also studies reporting inhibitory effects on tumor growth in an *in vivo* tumor model following MSC injection, using different approaches from ours [46,47]. The dose of MSCs delivered and the timing of injection have been highlighted as determining factors in the promotion or inhibition of tumor growth [28].

Although the mechanisms behind homing are not yet fully understood, the most likely cause of preferential migration is the release of chemotactic gradients from tumors. MSCs have a wide range of chemokine and cytokine receptors on their cell surface, which respond functionally to their ligands *in vitro*, whereas *in vivo*, their modification implies changes in migration behavior [48-52].

Some authors have shwon that MSCs secrete a large panel of chemokines such as CXCL12, CCL2, and CCL5, which implies activation of the MAPK, FAK, and STAT signaling pathways, and the induction of biological responses [48,49]. Moreover, tumors produce a wide range of chemokines and cytokines, which may act as ligands for MSC receptors [35,53]. Of the different pairs of receptor/ligand described as responsible for migration, the CXCL12/CXCR4 pair should be highlighted. This has been studied in numerous works, both *in vitro* and *in vivo,* and using different tumor models or MSCs sources [35,49-51,54-59]. Loss of expression in MSC´s receptors [48] implies a loss in cell-migration ability, indicating the great importance of these axes in MSC migration.

The relationship between homing and the inflammatory state has been assessed, and studies have been conducted in which BM-hMSCs were pre-treated with factors involved on inflammation (such as tumor necrosis factor, MMP2, CXCL12 and CCL5) [49]. But some studies were contradictory [35,50], which it could be due to the variation in donors or cell-culture conditions (confluence, hypoxia, and passages) [60].

Our results show that, in general, when MSCs engraft into tumors, migration-related gene expression is decreased (whereas hiPSCs exert an increase) except for the CXCL12/CXCR4 axis, causing a significant depletion in the expression of both markers when MSCs are present. The exceptions were hiPSCs, in which CXCR4 expression increased. The reason for this may be that CXCL12 expression is also linked to more immature cell fractions, with higher expression in less committed stages of differentiation, that is, in cells closer to the embryonic state [61]. With regard to hiPSCs, this study and another previous work [62] are the first to highlight their *in vivo* migration ability to and long-term engraftment into tissue damage areas.

Besides the release of chemotactic gradients from the tumors, other explanations for MSCs migration could be the hypoxic conditions produced by tumor cells, which may cause MSCs to increase the expression of migratory signals [63], and thus confer the ability to cross the biological barriers [59]. However, in our study, a direct correlation between the migration patterns and the absence of oxygen does not seem to exist (data not shown).

Finally, a recent dialogue mediated by exosomes between MSCs from the bone marrow and tumoral cells in patients with melanoma [64] has been shown. The degree of exosome release by the various MSCs could determine the migratory differences between those cells, depending both on their area of origin and also on their exosome targeting.

## Conclusion

This study clarifies the *in vivo* capacity of different types of MSCs and hiPSCs to migrate and engraft into a tumor model. The results reveal that the adult stem cells assayed were able to enter tumor sites and remain there, at least until the end of the experiments. Their different pluripotency pattern may also play a role in the differences between these cell types, in which higher imaging signals but also higher tumor growth were obtained in those with highest expression of pluripotency genes. Moreover, MSCs and hiPSCs also exerted different migration-related gene profiles in tumors, which could be attributed to the anti-inflammatory effect exerted by MSCs, thereby diminishing expression of the migration marker. Our promising data support the importance of MSCs as therapeutic gene carriers in anti-tumor treatments, and highlight the importance of choosing the most suitable lineage for specific tumor lesion. Further understanding of the role of MSCs in tumors and the promising possibility to use them as therapeutic gene vehicles may lead to potentially fruitful treatment approaches.

## Abbreviations

AdhNIS: Ad10 Adenoviral vector containing human sodium iodine symporter gene; APC: Activated protein C; BM-hMSC: Bone marrow-derived human mesenchymal stem cell; CCL2: Chemokine (C-C motif) ligand 2; CCL5: Chemokine (C-C motif) ligand 5; CXCL12: Chemokine (C-X-C motif) ligand 12; CXCR4: C-X-C chemokine receptor type 4; FACS: Fluorescence-activated cell sorting; FBS: Fetal bovine serum; FITC: Fluorescein isothiocyanate; FOV: Field of view; GADPH: Glyceraldehyde-3-phosphate dehydrogenase; hAFMSC: Human amniotic fluid mesenchymal stem cell; hAMC: Human amniotic membrane mesenchymal stem cell; hASC: Human adipose-derived stem cell; hEESC: Human epithelial endometrium-derived stem cell; hESSC: Human stromal endometrium-derived stem cell; hiPSC: Human induced pluripotent stem cell; hNIS: Human sodium iodine symporter; HPF: High-power field; HSD: Honestly significant difference; ICEp: Endometrial epithelial stem cell lines; ICEs: Endometrial stromal stem cell lines; MCP-1: Monocyte chemotactic protein-1; MMP: Matrix metalloproteinase; MMP-2: Matrix metalloproteinase-2; MOI: Multiplicity of infection; MRI: Magnetic resonance imaging; MSC: Mesenchymal stem cell; PE: Phycoerythrin; PerCP: Peridinin chlorophyll protein complex; pFU: Plaque-forming unit; qPCR: Quantitative polymerase chain reaction; PBS: Phosphate-buffered saline; RANTES: (Regulated on Activation, Normal T cell Expressed and Secreted); RARE: Rapid acquisition with relaxation enhancement; RT: Reverse transcription; SC: Subcutaneous; SDF-1: Stromal cell-derived factor-1; SI: Signal intensity; SPECT-CT: Single-photon emission computed tomography/X-ray computed tomography; SPIO: Super-paramagnetic iron oxide; TE: Echo time; TR: Repetition time; VOI: Volumes of interest.

## Competing interests

The authors indicate no competing financial interests.

## Authors’ contributions

CB-L participated in the design of the study, performed most of the experimental procedures, collected data, made the calculations, and participated in statistical analysis and in manuscript writing. GM participated in data analysis and interpretations, statistical analysis, and manuscript writing. DO and JB participated in the SPECT experiments. CS and IC carried out the collection and characterization of hEESCs and hESSCs. MI and JCR participated in the *in vitro* studies. PL-L performed MRI imaging acquisition. MQ participated in the *in vivo* studies. PM-D was the responsible of the conception and design of the study and participated in the *in vivo* studies, coordinated the study, and reviewed and corrected the manuscript. All authors read and approved the manuscript.

## Authors’ information

CB-L is a pre-doctoral staff member at the Instituto Aragones de Ciencias de la Salud-IIS Aragón, Zaragoza, Spain. GM is a post-doctoral fellow at the Instituto Aragones de Ciencias de la Salud-IIS Aragón, Zaragoza, Spain. DO is a visiting teacher at the Department of Medical Biochemistry and Microbiology, Uppsala University, Sweden. JB is a laboratory technician at the Queen Mary University of London, UK. CS is Full Professor of Obstetrics and Gynecology at the University of Valencia (Spain) and Scientific Head of the IVI. IC is Principal Investigator at IVI Foundation, Valencia, Spain. MI is Professor of Biochemistry and Dean of Faculty of Health Sciences at the University Francisco de Vitoria, Spain. JCR is Head of the Viral Vectors Unit at the Centro Nacional de Investigaciones Cardiovasculares, Spain. PL-L and MQ are Principal Investigators at the Instituto de Investigaciones Biomédicas Alberto Sols, Spain. PM-D is ARAID Foundation Principal Investigator, professor at University Francisco de Vitoria, Spain, and Head of the Gene and Therapy Group at the Instituto Aragones de Ciencias de la Salud-IIS Aragon, Zaragoza, Spain.

## Pre-publication history

The pre-publication history for this paper can be accessed here:

http://www.biomedcentral.com/1741-7015/11/139/prepub
